# Work, race and breastfeeding outcomes for mothers in the United States

**DOI:** 10.1371/journal.pone.0251125

**Published:** 2021-05-05

**Authors:** Margaret D. Whitley, Annie Ro, Anton Palma

**Affiliations:** 1 Program in Public Health, University of California, Irvine, Irvine, CA, United States of America; 2 Institute for Clinical and Translational Science, University of California, Irvine, Irvine, CA, United States of America; Christiana Care/University of Delaware, UNITED STATES

## Abstract

**Background:**

In the United States, mothers’ employment status and occupation are related to breastfeeding. However, it is unclear whether not working leads to longer breastfeeding duration even when compared to professional/managerial jobs, which tend to accommodate breastfeeding better than service/manual labor jobs. Furthermore, occupation and breastfeeding are racially patterned, and it is possible that race could moderate the relationships between mother’s work and breastfeeding.

**Methods:**

Using data from the Panel Study of Income Dynamics, we modeled breastfeeding duration based on mother’s employment/occupation (not working, professional/managerial work, or service/labor work) during the first 6 months postpartum, as well as mother’s race (White, Black or other) and other potential confounders. We used zero-inflated negative binomial regression models and tested an interaction between employment/occupation type and race. Predictive margins were used to compare breastfeeding duration among subgroups.

**Results:**

Mothers working in service/labor occupations had the shortest breastfeeding duration of the three employment/occupation groups, and there was no significant difference in duration between not working and professional/managerial occupation. White mothers had longer breastfeeding duration than Black mothers on average. When we included an interaction between employment/occupation and race, we found that among White mothers, non-working mothers breastfed the longest, while mothers in service/labor work breastfed for the shortest duration, but among Black mothers, mothers in professional/managerial work breastfed for longer than mothers in the other two work categories.

**Discussion:**

Race moderated the relationship between employment status/occupation type and breastfeeding such that, for White mothers, not working was the most advantageous circumstance for breastfeeding, in line with traditional work-family conflict theory. In contrast, for Black mothers, professional/managerial work was the most advantageous circumstance. These findings support the idea of the Market-Family Matrix, which allows that different work scenarios may be more or less advantageous for parenting behaviors like breastfeeding, depending on mothers’ circumstances.

## Introduction

### Empirical background

Breastfeeding is beneficial to the health of mothers and children [1–3]. For this reason, health organizations recommend exclusive breastfeeding until infants reach 6 months of age, and continued breastfeeding along with supplemental feeding until at least 12 months [2, 4]. In the United States (US), 79% of new mothers initiate breastfeeding, yet only 20% exclusively breastfeed until 6 months [5]. Breastfeeding behaviors can be influenced by employment status and occupation type [6]. For instance, mothers who do not work breastfeed longer on average than those who do work, especially those who work full-time [7–9]. Returning to work sooner after giving birth is associated with shorter breastfeeding duration [10, 11]. Over 25% of new mothers are working by the time the infant reaches three months [12], and issues with work or school are common reason for breastfeeding cessation [13]. Mothers who work in jobs that are hazardous, inflexible, lack parental leave benefits and/or do not have lactation accommodations tend to breastfeed for shorter duration [14–20]. Mothers working non-professional jobs, such as administrative, service and manual labor jobs also have shorter breastfeeding duration [11, 21–24], possibly because mothers working in those occupations experience more work-related barriers to breastfeeding, like not having time or an appropriate place to pump [25].

Given that working exposes mothers to many barriers to continued breastfeeding [25], mothers who do not work might be expected to breastfeed longer, even compared to mothers in relatively accommodating jobs. However, the literature presents a mixed picture. A New Hampshire study that examined breastfeeding by employment and industry category found that working mothers were less likely than non-working mothers to breastfeed at least four months regardless of industry category [24]. A California study that compared non-working mothers with mothers from three occupation groups found no difference in breastfeeding initiation rates, after accounting for other socioeconomic variables [26]. To our knowledge only two studies examined this question using US-wide data and using breastfeeding duration, as opposed to initiation, as an outcome. Kimbro and colleagues, using a racially diverse and mostly low-income sample of US births from 1998–2000, found that women who were not working breastfed longer than those in administrative or manual work, but not those in other occupations [21]. Visness and colleagues, using a representative sample of US births from 1988, found that non-working women breastfed longer than working women, regardless of the type of occupation, though among Black women this relationship was not consistent [22]. These studies established that the relationship between work and breastfeeding may vary considerably by occupation and by race.

There are persistent disparities in breastfeeding in the US by mother’s race: 64% of Black mothers initiate and 14% breastfeed exclusively at 6 months, compared to 82% and 23% among White mothers [5]. Race-based differences in breastfeeding have been linked to factors at many levels, including mothers’ beliefs and knowledge about breastfeeding [27–29], family norms [30], exposure to stressful life events [31, 32], healthcare interactions [30] and insurance [33], formula advertising and promotion [32, 34, 35], issues related to work and school [7, 36], and norms and policies at the state and region level [37]. Race is a social construct, and it relates to breastfeeding only because it determines mothers’ and infants’ exposure to other causal factors [38, 39]. Differences in breastfeeding outcomes between White and Black mothers have arisen in large part because of structural racism, both current and historical [38, 40, 41]. For instance, that Black women have more negative perceptions of breastfeeding and positive perceptions of formula feeding than White women [27, 42, 43], has been linked to lingering stigma and trauma from the slavery-era history of forced wet nursing and separation from one’s own infant [34, 44, 45]. Louis-Jacques and colleagues identified labor conditions as a major historical antecedent of current breastfeeding disparities [35]. In the present study we examine how present-day labor conditions continue to be intertwined with breastfeeding disparities between Black and White mothers in the US.

Occupation type, like breastfeeding behaviors, is racially patterned. Longstanding disparities in educational opportunities, hiring, firing and layoff practices and other structural barriers have greatly advantaged White Americans and disadvantaged Black Americans in their work opportunities [46–48]. White women, for example, are more likely to work in management or professional positions than Black women, while Black women are more likely to work in service occupations or transportation, in jobs that offer less flexibility [49, 50] and jobs without paid maternity leave [51]. Also, within a given occupation, Black mothers may face additional barriers to breastfeeding than White mothers [52], such as discriminatory treatment from a supervisor on the basis of their race [52–56].

### Theory

We consider two distinct theories specific to work and family that paint distinct pictures of how employment and occupation may relate to breastfeeding and how this might vary by race. On the one hand, the Work-Family Conflict theory suggests that women’s work responsibilities can conflict with responsibilities related to breastfeeding, an activity tied to the family realm [57]. In addition, the concept of Intensive Mothering implies that idealized child rearing requires large amounts of time and resources from mothers, often at the expense of their professional priorities [58, 59]. Both of these suggest that mothers who do not work would have an advantage in breastfeeding over mothers who work, regardless of mother’s occupation or race.

On the other hand, Dow’s Market-Family Matrix framework posits that work and family responsibilities do not necessarily have to be in conflict, and that they can integrate in beneficial ways if circumstances make that possible [60, 61]. This framework grew from qualitative research with middle class Black mothers the US which showed that, whereas White mothers held positive perceptions of not working while their children were young [62], Black mothers were more likely to have negative perceptions about not working, and more positive perceptions of being a working parent [60, 63]. For instance, Dow described how the middle class African American mothers in her study tended to believe that “working is a duty of motherhood and does not detract from one’s identity as a mother,” (page 165). She contrasted that with the notion, based on beliefs among middle class White women, that working necessarily impedes a woman’s ability to be a good mother [60]. Extending Dow’s Market-Family Matrix framework to breastfeeding, we consider that employment status could be differentially associated with breastfeeding outcomes, and that whether employment is associated with more or less breastfeeding may vary based on mother’s race and on the kind of job they hold.

In sum, existing theories about work-family integration suggest two distinct possibilities about maternal work and breastfeeding outcomes: one, that maternal employment would consistently be associated with less breastfeeding, or two, that there may be instances when working mothers have comparable or better breastfeeding outcomes than mothers who do not work, particularly among professional Black mothers. There is little existing research that has comprehensively examined race as well as occupation type in connection with breastfeeding outcomes, but older evidence may support the latter perspective. Visness and colleagues’ found that for Black mothers, not working did not confer an advantage for breastfeeding duration as consistently as it does among White mothers [22]. However, the births included in the Visness paper occurred in 1988, and the prevalence and duration of breastfeeding [64] and of maternal employment [12] in the US have increased substantially since then. New evidence is needed to reexamine these relationships.

### Study objectives

To fill these gaps in the literature and contribute to theory around work, family, race and health behaviors, we used a national data set of US families, including births from 2008–2013, to examine two questions. First, we examined how employment status and occupation type were related to breastfeeding duration, particularly when included alongside race in a multivariate model. We hypothesized that not working would be associated with the longest average breastfeeding duration compared to both professional/managerial occupations and to service/labor occupations. We also hypothesized that among those who work, a professional/managerial occupation would be associated with longer breastfeeding duration than working in a service/labor occupation.

Second, we wanted to explore whether employment status and occupation type related to breastfeeding duration differentially for White versus Black mothers. We hypothesized that race would moderate the relationship between employment/occupation type and breastfeeding, and we considered different possible scenarios for that relationship. In one scenario, not working (compared to working) would be associated with more breastfeeding among White mothers, but with less breastfeeding among Black mothers, particularly when compared to working in professional jobs. This is based on prior research [22] and on qualitative work and theory [60]. It could be the result of greater social support for working mothers in Black versus White communities, as well as differential selection into employment among mothers of young children, in part because of different preferences [60]. An alternative scenario would be that not working is associated with more breastfeeding among both groups of mothers, regardless of the occupation of mothers in the comparison groups, and that the difference is larger among Black mothers than among White mothers. This could happen because working creates additional barriers to breastfeeding for Black mothers than it does for White mothers, such as discriminatory treatment at work [53].

Findings with regard to these questions would inform theoretical understandings about the interplay between work, family and race. They would also provide newer quantitative estimates of the degree of these relationships from a sample of US mothers. Also, the findings could help inform public health policy efforts to increase breastfeeding among all US mothers and reduce breastfeeding disparities [65].

## Methods

### Data set

The Panel Study of Income Dynamics is a longitudinal, nationally representative survey of US families conducted at the University of Michigan, which has followed multiple generations of the same families since as early as 1968 [66, 67]. The survey includes extensive information about socioeconomic variables, family characteristics and other topics, and refresher samples have been added to the dataset since its inception to increase representativeness. The Panel Study of Income Dynamics collected Child Development Supplement data in 2014 to obtain more detailed information on development, health and other topics for the children of the main sample members. We linked data on children’s health outcomes, including breastfeeding, from the 2014 Child Development Supplement with data on their biological mother’s sociodemographic and work characteristics from the main study (waves 2007 through 2015). Data were collected using computer-assisted telephone interviews. Most of the data are publicly available online [67].

### Inclusion criteria

Respondents had to be the biological mother of a child in the 2014 Child Development Supplement and have complete information on employment and occupation for the five months after the month when the child was born. Because Panel Study of Income Dynamics respondents were labeled only as male or female, we have no information about other gender identities [68].

Of the 4,333 children in the Panel Study of Income Dynamics’ Child Development Supplement, 3019 were excluded because they were over age 5 at the time of data collection. Of the remaining 1314, an additional 6 were excluded because we could not link the child to their biological mother. Of the remaining 1308, 338 were excluded due to missing information on one or more key variables. The most commonly missing data was employment and occupation type for the first six months postpartum (n = 283). After these exclusions, we had an analytic sample of n = 970 cases.

Because there were siblings among the 970 children in the analytic sample, there were 802 unique mothers included. For all variables except for race, we used responses specific to the time period when that child was born. For mother’s race, we used the most recent response available. Sensitivity analyses were used to address potential clustering effects among children with the same biological mother.

Our study was reviewed by the University of California, Irvine Human Research Projections Program and determined to not qualify as human subjects research. The data that we received and used for this secondary analysis did not contain identifying information for study participants. To note, the researchers who originally collected the Panel Study of Income Dynamics data obtained informed consent from all study participants [67].

### Measures

#### Breastfeeding initiation and duration

Respondents were asked if participating children age 5 years or less were breastfed at all, and if so, at what age (in months) did the child stop breastfeeding. Breastfeeding duration ranged between 1 and 60 months; responses of 13 months or more were top-coded to 12 months, given the recommendations and evidence about breastfeeding for that duration [2]. All mothers who were still breastfeeding at the time of data collection had already breastfed more than 12 months, so we did not have any instances of censored breastfeeding data.

#### Mother’s race

Respondents could select up to three races; we used the first race mentioned. We collapsed Latino/Hispanic, Asian, Native Hawaiian or Pacific Islander, and Other into a single category, Other Races, and left White and Black as additional categories.

#### Occupation

In all waves of the survey, adults reported employment status every month. We obtained the mother’s employment status for the first through fifth months following the month when the child was born. Mothers who reported either not working or being unemployed for all five months were categorized as not working for this analysis. For those who reported working at least one of those five months, whether in their primary or secondary job, we considered them employed in the analyses and obtained the occupation code associated with that job. Occupation codes were in 3-digit, US Census 2000 format [66, 69]. We classified codes 01–354 as professional/managerial occupations–this included Education, Training, and Library Occupations (codes 220–255) and Healthcare Practitioners and Technical Occupations (300–354). We categorized codes 360 through 983 as service/manual labor occupations; these included Office and Administrative Support Occupations (500–593) and Personal Care and Service Occupations (430–465).

Self-employment was categorized like other jobs. Mothers whose employment status did not fit these categories, for instance because they were students and did not report working, were excluded from analysis because they did not have an occupation code. The survey did not specifically assess maternity leave. If a mother was on maternity leave for the five months after giving birth (or left her job) and described herself as not working in the survey, then she was classified as not working. However, if a mother had maternity leave for any length of time but still listed herself as working for a given job during at least one of those five months, then she was categorized as working in that job.

#### Covariates

We controlled for other variables that were shown to be associated with maternal race and breastfeeding behaviors in the literature and in our bivariate analyses. Specifically, we controlled for maternal age at delivery (continuous) [70, 71]; educational attainment [71–73] dichotomized at 16 or more years, approximately equivalent to having a bachelor’s degree; mother’s marital status [74, 75] from a variable classifying adults as either the wife/spouse or, if not married, the “head of house”; and whether the child was born with low birth weight [76, 77], defined as below 88 ounces (5.5 lbs).

### Analysis

#### Descriptive statistics

We conducted descriptive statistics for all variables, specifically, weighted means and standard deviations for continuous variables and weighted proportions for categorical variables, for the overall sample and stratified by race and by employment status. All statistical analyses were conducted in Stata 16.1 MP. Alpha of 0.05 was used to determine statistical significance.

#### Multivariate models

We used zero-inflated negative binomial regression models to estimate breastfeeding duration ratios, where breastfeeding duration was modeled as a count of the total number of months that the child was reported to have been breastfed. This modeling approach was chosen since breastfeeding involves both the choice to initiate breastfeeding and to maintain breastfeeding once initiated. Although zero-inflated negative binomial models can allow for different sets of covariates in the duration and initiation models, we used the same set of variables based on the literature and our bivariate analyses which suggested that they are related in similar ways to both breastfeeding outcomes. We conducted a Vuong test to confirm that there was overdispersion of zero values and that a zero-inflated model was appropriate. All models accounted for sampling weights.

We created two nested multivariate zero-inflated negative binomial regression models, both predicting breastfeeding duration and initiation. The first model included the main effects for maternal race and employment/occupation type, adjusted for covariates, and the second model included those same variables as well as an interaction between race and occupation type. In both models, race was modeled using a categorical variable with three categories: White race (reference group), Black race and Other races. White was used as the reference group because it is the largest race group in this sample. Occupation type was also modeled as a categorical variable with three categories: not working (reference group), professional/managerial jobs and service/labor jobs. Not working was used as the reference group so that we could compare outcomes for mothers exposed to distinct employment situations to mothers who are not employed.

From these models, we estimated predicted breastfeeding duration by maternal race and occupation and graphed those predictions for the models without and with an interaction term. We used contrasts of marginal linear predictions to test which pairwise comparisons of occupation groups by race, and of race by occupation groups resulted in statistically significant differences.

We accounted for the sampling design by using the main child sample weights for the Child Development Supplement (based on child’s sex, birth year, race/ethnicity and geographic region), as well as the stratum and cluster weights for the main Panel Study of Income Dynamics survey. It should be noted that the weights were not designed for our specific subsample (e.g. mothers of children younger than 5).

### Sensitivity analyses

We used alternative modeling approaches to account for potential clustering effects for children born to the same mother. These included running the models with only one child per mother and using multilevel models with a random intercept for the mother. We also ran the models with robust standard errors.

## Results

### Descriptive statistics

The characteristics of the study sample are shown in Table 1. Overall, 71% of mothers initiated breastfeeding, and this was notably lower for Black mothers (54%) and those working in service or labor occupations (63%). Among those who breastfed, average duration was 6.9 months; when stratified by race, breastfeeding duration was longest among White mothers at 7.3 months, followed by mothers of other races at 6.3 months, while Black mothers had shortest average duration, with 5.1 months. By employment and occupation type, mothers who did not work or who worked in professional/managerial occupations had similar duration, 7.3 and 7.4 months respectively, while those in service/labor occupations had 5.9 months average duration.

**Table 1:** Weighted sociodemographic characteristics, health outcomes and occupational characteristics for analytic sample of mothers and infants.

	Total n = 970	By employment status/occupation type			By race		
		Not working	Managerial/professional occupation	Service/manual labor occupation,	White	Black	Other
		n = 340	n = 252	n = 378	n = 510	n = 354	n = 106
**Mother’s socio-demographic characteristics**							
** Race**							
** White, %**	69.1	69.0	76.9	62.4			
** Black, %**	13.6	13.5	8.5	18.2			
** Other races, %**	17.3	17.6	14.6	19.4			
** Age, mean (SD) years**	29.1 (5.4)	27.9 (5.7)	31.0 (4.4)	28.7 (5.6)	29.7 (5.2)	26.5 (4.8)	28.9 (6.1)
** Education (bachelors degree or higher), %**	42.0	28.0	76.3	25.2	50.4	19.2	26.1
** Mother is not married, %**	15.1	13.5	8.8	22.1	9.5	51.8	8.5
**Health outcomes and breastfeeding behaviors**							
** Breastfeeding behaviors**							
** Child initiated breastfeeding, %**	71.4	71.4	80.0	63.6	74.0	54.1	74.4
** Breastfeeding duration, mean (SD) months**^**a**^ **(Among the n = 628 who initiated)**	6.9 (4.0)	7.3 (4.1)	7.4 (3.9)	5.9 (3.9)	7.3 (4.0)	5.1 (3.4)	6.3 (4.2)
** Infant born with low birth weight, %**	6.2	6.5	5.6	6.3	6.3	8.9	3.5
**Mother’s employment and occupation, first 6 months postpartum**							
** Not working, %**	34.2				34.2	34.0	34.8
** Managerial/professional occupation, %**	30.9				34.4	19.4	26.1
** Service/manual labor occupation, %**	34.9				31.5	46.6	39.2

^a^Breastfeeding duration was top-coded at 12 months.

SD = standard deviation.

Proportions (%), means, and standard deviations take Panel Study of Income Dynamics survey weights into account.

Approximately two thirds of mothers in the sample were employed during the first six months postpartum, with 35% in service/manual labor occupations and 31% in managerial/professional occupations. Among Black and White mothers, 34% reported not working, for mothers of other races, 35% reported not working. White mothers were overrepresented for managerial/professional occupations, as 34% of those workers were White, while Black women were overrepresented for service/manual labor occupations, among whom 47% were Black.

### Work and breastfeeding duration

The results of the regression model without the occupation by race interaction are provided in Table 2. We did not find any significant differences in odds of breastfeeding initiation by occupation or race.

**Table 2:** Breastfeeding duration and initiation by mother’s employment status/occupation type and race.

	Model 1		Model 2	
**Breastfeeding initiation**	**Ratio**	**(95% CI)**	**Ratio**	**(95% CI)**
Occupation type (Not working = ref)				
** **Professional/managerial	1.146	(0.642, 2.046)	0.942	(0.463, 1.920)
** **Service/labor	0.747	(0.438, 1.274)	0.735	(0.379, 1.425)
Race (White = ref)				
** **Black race	0.620	(0.374, 1.028)	0.641	(0.278, 1.476)
** **Other race	1.294	(0.750, 2.232)	0.926	(0.325, 2.638)
Occupation type * Race (White and not working = ref)				
** **Black race & professional/managerial			1.114	(0.260, 4.784)
** **Black race & service/labor			0.868	(0.320, 2.359)
** **Other race & professional/managerial			5.860	(0.429, 79.967)
** **Other race & service/labor			1.170	(0.192, 7.142)
**Breastfeeding duration**	**Duration Ratio**	**(95% CI)**	**Duration Ratio**	**(95% CI)**
Occupation type (Not working = ref)				
** **Professional/managerial	0.953	(0.815, 1.115)	0.904	(0.762, 1.072)
** **Service/labor	0.843*	(0.716, 0.993)	0.795*	(0.663, 0.954)
Race (White = ref)				
** **Black race	0.783*	(0.658, 0.933)	0.562*	(0.444, 0.712)
** **Other race	0.865	(0.744, 1.004)	0.812	(0.590, 1.118)
Occupation type * Race (White and not working = ref)				
** **Black race & professional/managerial			1.720*	(1.101, 2.686)
** **Black race & service/labor			1.546*	(1.020, 2.343)
** **Other race & professional/managerial			1.083	(0.672, 1.746)
** **Other race & service/labor			1.122	(0.670, 1.877)

CI = Confidence Interval. Ref = Reference category.

*P-value <0.05.

Both models are zero-inflated negative binomial models (ZINB) with n = 970 participants. Coefficients displayed here have been exponentiated. The ratios in the top half of the table represent the odds of initiating breastfeeding; we obtained these by exponentiating and then inverting the original coefficients from the ZINB model. The ratios in the bottom half represent ratios for breastfeeding duration. Models included survey weights. Both models control for mother’s age in years at time of child’s birth (range 15.6 to 45.8 years), mother’s educational attainment (college graduate versus not), mother’s marital status, and whether the infant is was born with low birth weight or not).

**Fig 1:**
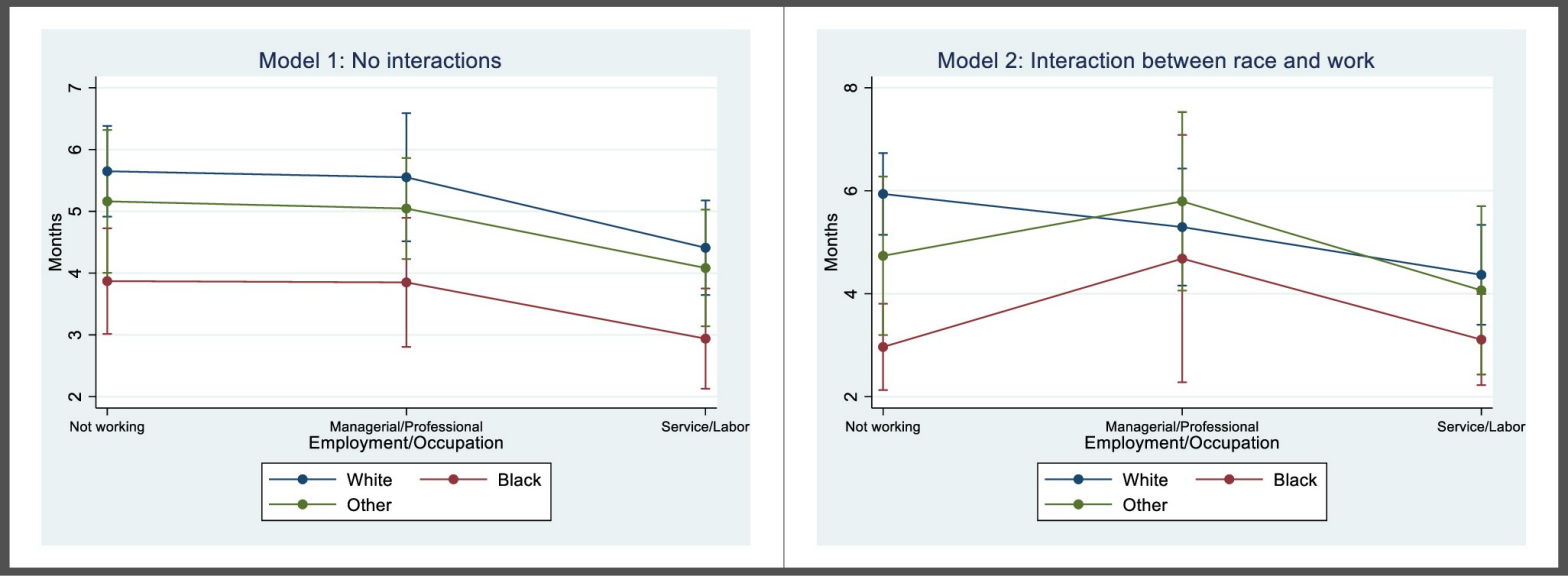
Predicted breastfeeding duration by mother’s employment status/occupation type and race. The predicted margins for breastfeeding duration are based on two adjusted zero-inflated negative binomial regression models: Model 1 predicted breastfeeding duration based on mother’s employment status/occupation type and race with no interaction term, while Model 2 included an interaction between race and employment status/occupation type. Predicted values and confidence intervals are shown in S1–S3 Tables.

Mothers in a service/labor occupation had the shortest average breastfeeding duration of the three groups. After accounting for potential differences in breastfeeding initiation by work status, in Model 1 we found that working in a service/labor occupation was associated with significantly shorter breastfeeding duration compared to not working during the first 6 months postpartum (ratio [95% CI] = 0.84 [0.72, 0.99]), after accounting for potential confounders. Working in a professional/managerial occupation was not significantly different from not working (ratio [95% CI] = 0.95 [0.82,1.12]). These findings indicate that women working in a service/labor occupation stopped breastfeeding 16% earlier than their non-working counterparts. These relationships were also apparent in the predicted duration by race in Fig 1, where breastfeeding duration was similar for mothers who did not work and those in professional/management jobs, and notably lower for those in service/labor jobs.

Model 1 also showed that Black mothers had breastfeeding duration that was roughly three-quarters that of White mothers (ratio [95% CI] = 0.78 [0.66, 0.93]). In other words, after accounting for potential differences in breastfeeding initiation by race, Black mothers stopped breastfeeding 22% earlier than their White counterparts. Women of other races did not have significantly different average breastfeeding duration compared to White women in the fully adjusted model. This appears in Fig 1 as well, where predicted breastfeeding duration for Black mothers is the lowest for all groups, consistently below four months, while White mothers had the longest duration, followed closely by mothers of other races.

Of the other covariates included in Model 1, older age was positively associated with duration, while the mother being not married was significantly and negatively associated with duration (see S1 Table).

#### Interaction model

Model 2 included an interaction between employment/occupation type and race. The interactions for Black versus White race and both occupation groups were statistically significant, indicating that the relationship between employment/occupation type and breastfeeding duration differed by race. There was a strong interaction effect for Black mothers in professional jobs compared to white mothers who did not work, ratio [95% CI] = 1.72 [1.10, 2.69], and for Black mothers in a service/labor job compared to white mothers who did not work, ratio [95% CI] = 1.55 [1.02, 2.34]. This was apparent in the predicted duration values shown in Fig 1 (and S2 Table). For White mothers, not working was still associated with the longest duration (predicted duration [95% CI] = 5.94 months [5.15, 6.73]), followed by managerial/professional work (duration [95% CI] = 5.29 months [4.16, 6.43]), with service/labor workers having the shortest average duration (duration [95% CI] = 4.37 months [3.40, 5.34]). However, for Black mothers, breastfeeding duration was longest among those working in a managerial/professional occupation (predicted duration [95% CI] = 4.68 months [2.28, 7.08]), and shorter for mothers who do not work (duration [95% CI] = 2.97 months [2.13, 3.80]) and who work in a service/labor occupation (duration [95% CI] = 3.11 months [2.23, 3.99]). A similar if attenuated pattern emerged for women of other races, where managerial/professional work was associated with the longest average breastfeeding duration.

Contrasts of marginal linear predictions showed that the lines representing breastfeeding duration across the three occupation groups were not parallel for Black and White mothers (p = 0.029), and in particular among mothers who did not work, predicted duration was significantly longer for White mothers than Black mothers (p<0.001). Within race groups, predicted duration was significantly different comparing managerial/professional jobs versus not working among Black mothers (p = 0.030) and comparing service/labor jobs versus not working among White mothers (p = 0.015).

### Sensitivity analyses

The models that accounted for potential clustering among children born to the same mother did not produce notably different results. S3 Table shows the results of interaction models for breastfeeding initiation and duration without and with random effects for siblings born to the same mother. The relationships between race, occupation and breastfeeding outcomes are consistent in terms of direction and statistical significance even when potential clustering among siblings was included. One exception was that in the breastfeeding duration models that accounting for clustering among siblings, the coefficient for the interaction between Black (vs White) race and professional/managerial work (vs not working) was no longer statistically significant, although it remained similar in magnitude. Conversely, the coefficient for the interaction between Black (vs White) race and service/labor work (vs not working) became statistically significant in the model that accounted or sibling clustering; the magnitude of this coefficient was also similar compared to the original model.

## Discussion

### Main finding

We found that among US mothers, working in a service/labor job was associated with about one month less breastfeeding compared to not working or professional/management work. When we considered race as a potential moderator, we found that while not working was associated with the longest breastfeeding duration among White mothers, working in a professional/management job was associated with the longest breastfeeding duration among Black mothers.

Our hypothesis that not working would be associated with longer duration overall was supported, but only in comparison to service/labor occupations, which are typically less accommodating of breastfeeding [25]. Not working was not significantly different from professional/managerial work, which is more likely to accommodate breastfeeding. This aligns with the findings of Kimbro and colleagues, who found that working in an administrative or manual job was associated with one-third higher odds of quitting breastfeeding than not working, while they found no significant differences between not working and the other occupations [21]. Our findings also corroborate literature showing that professional and managerial jobs are associated with longer breastfeeding duration than administrative, manual or service jobs [11, 23].

When we considered differences by race, the presumptive advantage of not working on breastfeeding only held for White women. Not working was *not* associated with an apparent advantage in breastfeeding duration among Black women or women of other races; in fact, among Black women, working in professional/managerial jobs was associated with the longest breastfeeding duration on average across our three occupation categories. This contrasted with the results of Visness and colleagues in that they found not working to be advantageous for breastfeeding duration for White and Black mothers. However, they found this advantage to be stronger and more consistent among White mothers than among Black mothers [22].

There are multiple possible explanations for the finding that, among Black mothers, the longest breastfeeding duration occurred among those in professional/management work, while among White mothers, the longest duration occurred among those who did not work. In general, this aligns with the thrust of Dow’s Market-Family Matrix framework, that paid work and motherhood responsibilities can sometimes be mutually beneficial and sometimes come into conflict [60]. Exposure to institutional racism [78] and the impact of breastfeeding policies [78] differentially affect women by race, and this could contribute to the moderating effect by race. Alternatively, there may be differential selection into not working that influenced breastfeeding among Black and White mothers. This selection could be driven by different preferences, ideals and concerns about staying at home versus working while one’s child is young.[60, 79] These differences in norms and preferences could feed into distinct decision-making processes about employment/occupation and breastfeeding for White and Black mothers. It is also possible that Black professional mothers who overcame racial adversity in the workplace were better resourced or had better health.

To note, even in the subgroups that had the highest breastfeeding rates, such as mothers in management/professional occupations, a large proportion of mothers were not meeting the six-month breastfeeding recommendation [2]. Even among the mothers in professional/management jobs, 20% did not initiate breastfeeding, and among those who did initiate, the average duration of 7.4 and a standard deviation of nearly 4 months indicated that many had duration far below the six month target. These findings align with the idea that greater support is needed for continued breastfeeding for all populations of women in the US [65].

### Strengths and limitations

This study had some limitations. The Panel Study of Income Dynamics Child Development Supplement did not collect data about whether breastfeeding was exclusive or not. The breastfeeding advantage associated with not working may be stronger if exclusive breastfeeding is the outcome of interest. In addition, prior research suggests Black mothers are more likely than White mothers to supplement with formula [80], so a focus on exclusive breastfeeding could show larger racial disparities. Also, we operationalized work experiences using broad groupings of occupation codes, and this meant that we could not capture certain differences within these broad occupational categories that are important for breastfeeding, like schedule flexibility [14], experiences specific to the worksite, or the unique pressures or stressors facing Black workers compared to White workers [52, 81]. This could mean that we are underestimating the role of work on breastfeeding outcomes. Health selection into employment could complicate the relationship between not working and breastfeeding [82]. Finally, we could not account in a rigorous way for maternity leave, which may be related to breastfeeding duration [19].

These limitations are counterbalanced with several strengths. Panel Study of Income Dynamics captured work data specifically for the period immediately following the child’s birth. This was an advantage over other studies that relied on occupation during pregnancy [25]. Also, we used breastfeeding duration as an outcome, which provided more granularity and more statistical power than a dichotomous outcome, e.g. whether or not mothers achieved six months of breastfeeding.

The present work adds additional information to the established positive relationship between socioeconomic status and breastfeeding duration [26, 71] by centering the unique role of occupational status [5]. Income, occupation, education are distinct but interrelated ways to measure socioeconomic status [83, 84]. In the present study, we were interested in occupation not as a measure of socioeconomic status or social class, but rather as a proxy for working conditions [85, 86] related to breastfeeding, such as barriers to taking lactation breaks and control over her schedule, that a mother might experience during working hours. Even after controlling for education, we found that occupation type was a significant predictor of breastfeeding duration. Future research should study whether occupation type mediates the relationship between income and breastfeeding duration. Also, future studies should examine whether other causal factors associated with education, income and occupation, such as quality of lactation care [87], may explain some of the relationship between occupation type and racial differences in breastfeeding.

## Conclusions

Our findings suggest it is important to take race into account when drawing broader conclusions about how work can affect parenting-related behaviors like breastfeeding. Particularly in the US, structural racism has had and continues to have a powerful impact on the parenting choices and work opportunities available to Black and White mothers. There are striking historical examples, particularly the legacy of slavery and of Black mothers being forced to nurse White infants, sometimes at the expense of feeding their own child [35, 44, 45]. Those factors continue to have ripple effects, and are layered on top of breastfeeding challenges Black mothers still may face, like racism on the job [52], as well as innovative resources and strengths present in their communities [60, 88, 89].

We found that the racial disparities in breastfeeding were smaller among women in managerial/professional occupations. However, there is a clear need to increase workplace supports for breastfeeding for all working women, and particularly those in labor and service jobs. Relevant interventions already exist [89–91], including lactation programs for hospital employees [92] and corporate initiatives to reduce work-family conflict and increase schedule control [93]. These could be implemented more broadly and should be tailored to Black mothers. Future work should consider specific working conditions like demands and controls [94] as well as union presence [95, 96] and explore how they relate to both race and breastfeeding. Selection into employment and occupation should be further examined, potentially using propensity score matching [9, 97]. Future research also needs to consider breastfeeding and work experiences among other groups of parents, in particular Latinx, Asian American and Native American/American Indian families, immigrants [26, 98], as well as trans-identified or other gender non-binary, breast or chest-feeding parents [68], and lesbian and gay parents [99].

## Supporting information

S1 Table Full set of multivariate models predicting breastfeeding duration and non-initiation by mother’s race and employment status/occupation type. (DOCX)

S2 Table Predicted breastfeeding duration based on mother’s employment status/occupation type and race. (DOCX)

S3 Table Sensitivity analyses to assess effects of clustering among siblings born to same mother. (DOCX)

## References

[1] Ip S, Chung M, Raman G, Trikalinos TA, Lau J. A summary of the Agency for Healthcare Research and Quality’s evidence report on breastfeeding in developed countries. *Breastfeed Med*. 2009;4(S1):S-17–S-30. 10.1089/bfm.2009.0050 19827919

[2] Eidelman AI, Schanler RJ. Breastfeeding and the use of human milk. *Pediatrics*. 2012;129(3):e827–e841. 10.1542/peds.2011-3552 22371471

[3] Victora CG, Bahl R, Barros AJ, et al. Breastfeeding in the 21st century: epidemiology, mechanisms, and lifelong effect. *Lancet*. 2016;387(10017):475–490. 10.1016/S0140-6736(15)01024-7 26869575

[4] World Health Organization. Infant and young child feeding. 2020; Available from: https://www.who.int/en/news-room/fact-sheets/detail/infant-and-young-child-feeding. Accessed Nov 3, 2020.

[5] Anstey EH, Chen J, Elam-Evans LD, Perrine CG. Racial and geographic differences in breastfeeding—United States, 2011–2015. *MMWR Morb Mortal Wkly Rep*. 2017;66(27):723. 10.15585/mmwr.mm6627a3 28704352 PMC5687589

[6] Roe B, Whittington LA, Fein SB, Teisl MF. Is there competition between breast-feeding and maternal employment? *Demography*. 1999;36(2):157–171. 10332608

[7] Ryan AS, Zhou W, Arensberg MB. The effect of employment status on breastfeeding in the United States. *Womens Health Issues*. 2006;16(5):243–251. 10.1016/j.whi.2006.08.001 17055377

[8] Attanasio L, Kozhimannil KB, McGovern P, Gjerdingen D, Johnson PJ. The impact of prenatal employment on breastfeeding intentions and breastfeeding status at 1 week postpartum. *J Hum Lact*. 2013;29(4):620–628. 10.1177/0890334413504149 24047641 PMC3835540

[9] Lubold AM. Breastfeeding and employment: A propensity score matching approach. *Sociol Spectr*. 2016;36(6):391–405.

[10] Chatterji P, Frick KD. Does returning to work after childbirth affect breastfeeding practices? *Rev Econ Househ*. 2005;3(3):315–35.

[11] Dagher RK, McGovern PM, Schold JD, Randall XJ. Determinants of breastfeeding initiation and cessation among employed mothers: a prospective cohort study. *BMC pregnancy and childbirth*. 2016;16(1):194. 10.1186/s12884-016-0965-1 27472915 PMC4966748

[12] Laughlin LL. Maternity leave and employment patterns of first-time mothers:1961–2008. US Department of Commerce, Economics and Statistics Administration; 2011.

[13] Ahluwalia IB, Morrow B, Hsia J. Why do women stop breastfeeding? Findings from the Pregnancy Risk Assessment and Monitoring System. *Pediatrics*. 2005;116(6):1408–12. 10.1542/peds.2005-0013 16322165

[14] Johnson KM, Salpini C. Working and nursing: navigating job and breastfeeding demands at work. *Community Work Fam*. 2017;20(4):479–96.

[15] Guendelman S, Kosa JL, Pearl M, Graham S, Goodman J, Kharrazi M. Juggling work and breastfeeding: effects of maternity leave and occupational characteristics. *Pediatrics*. 2009; 123(1):e38–e46. 10.1542/peds.2008-2244 19117845

[16] Jacknowitz A. The role of workplace characteristics in breastfeeding practices. *Women Health*. 2008;47(2):87–111. 10.1080/03630240802092357 18681102

[17] Spitzmueller C, Zhang J, Thomas CL, Wang Z, Fisher GG, Matthews RA, et al. Identifying job characteristics related to employed women’s breastfeeding behaviors. *J Occup Health Psychol*. 2018;23(4):457. 10.1037/ocp0000119 29756788

[18] Calnen G. Paid maternity leave and its impact on breastfeeding in the United States: an historic, economic, political, and social perspective. *Breastfeed Med*. 2007;2(1):34–44. 10.1089/bfm.2006.0023 17661618

[19] Whipps MDM, Honoroff J. Time off work after childbirth and breastfeeding supportive workplaces: associations with near-exclusive breastfeeding trajectory membership. *Womens Health Issues*. 2019;29(6):506–12. 10.1016/j.whi.2019.08.006 31628004

[20] Lauer EA, Armenti K, Henning M, Sirois L. Identifying barriers and supports to breastfeeding in the workplace experienced by mothers in the New Hampshire Special Supplemental Nutrition Program for Women, Infants, and Children utilizing the Total Worker Health framework. *Int J Environ Res Public Health*. 2019;16(4):529. 10.3390/ijerph16040529 PMC6406909 30781764

[21] Kimbro RT. On-the-job moms: work and breastfeeding initiation and duration for a sample of low-income women. *Matern Child Health J*. 2006;10(1):19–26. 10.1007/s10995-005-0058-7 16521055

[22] Visness CM, Kennedy KI. Maternal employment and breast-feeding: findings from the 1988 National Maternal and Infant Health Survey. *Am J Public Health*. 1997;87(6):945–50. 10.2105/ajph.87.6.945 9224174 PMC1380928

[23] Snyder K, Hansen K, Brown S, Portratz A, White K, Dinkel D. Workplace breastfeeding support varies by employment type: the service workplace disadvantage. *Breastfeed Med*. 2018;13(1):23–7. 10.1089/bfm.2017.0074 29185806

[24] National Institute for Occupational Safety and Health Surveillance Program, Institue on Disability Research. Supplement report: analysis of New Hampshire Pregnancy Risk Assessment Monitoring System (PRAMS) to better understand breastfeeding initiation and duration by industry category. 2020.

[25] Whitley MD, Ro A, Choi B. Workplace breastfeeding support and job satisfaction among working mothers in the United States. *Am J Ind Med*. 2019;62(8):716–26. 10.1002/ajim.22989 31168846 PMC8423352

[26] Heck KE, Braveman P, Cubbin C, Chavez GF, Kiely JL. Socioeconomic status and breastfeeding initiation among California mothers. *Public Health Rep*. 2006;121(1):51–9. 10.1177/003335490612100111 16416698 PMC1497787

[27] Jefferson UT. Breastfeeding exposure, attitudes, and intentions of African American and Caucasian college students. *J Hum Lact*. 2017;33(1):149–56. 10.1177/0890334416679384 28135485

[28] McCarter-Spaulding D, Gore R. Breastfeeding self-efficacy in women of African descent. *J Obstet Gynecol Neonatal Nurs*. 2009;38(2):230–43. 10.1111/j.1552-6909.2009.01011.x 19323720

[29] Bai Y, Wunderlich SM, Fly AD. Predicting intentions to continue exclusive breastfeeding for 6 months: A comparison among racial/ethnic groups. *Matern Child Health J*. 2011;15(8): 1257–64. 10.1007/s10995-010-0703-7 21057864

[30] McKinney CO, Hahn-Holbrook J, Chase-Lansdale PL, Ramey SL, Krohn J, Reed-Vance M, et al. Racial and ethnic differences in breastfeeding. *Pediatrics*. 2016;138(2):e20152388. 10.1542/peds.2015-2388 27405771 PMC4960721

[31] McLaughlin KA, Alvarez K, Fillbrunn M, Green JG, Jackson JS, Kessler RC, et al. Racial/ethnic variation in trauma-related psychopathology in the United States: a population-based study. *Psychol Med*. 2019;49(13):2215–26. 10.1017/S0033291718003082 30378513 PMC6494744

[32] Cricco-Lizza R. Infant-feeding beliefs and experiences of Black women enrolled in WIC in the New York metropolitan area. *Qual Health Res*. 2004;14(9):1197–210. 10.1177/1049732304268819 15448295

[33] Gurley-Calvez T, Bullinger L, Kapinos KA. Effect of the Affordable Care Act on breastfeeding outcomes. *Am J Public Health*. 2018;108(2):277–83. 10.2105/AJPH.2017.304108 29267066 PMC5846575

[34] Asiodu I, Flaskerud JH. Got Milk? A Look at Breastfeeding from an African American Perspective. *Issues Ment Health Nurs*. 2011;32(8):544–6. 10.3109/01612840.2010.544842 21767257

[35] Louis-Jacques AF, Marhefka SL, Brumley J, Schafer EJ, Taylor TI, Brown AJ, et al. Historical antecedents of breastfeeding for African American Women: from the pre-colonial period to the mid-twentieth century. *J Racial Ethn Health Disparities*. 2020;7(5):1003–12. 10.1007/s40615-020-00727-5 32124420

[36] Johnson A, Kirk R, Rosenblum KL, Muzik M. Enhancing breastfeeding rates among African American women: A systematic review of current psychosocial interventions. *Breastfeed Med*. 2015;10(1):45–62. 10.1089/bfm.2014.0023 25423601 PMC4307211

[37] Hannan A, Li R, Benton-Davis S, Grummer-Strawn L. Regional variation in public opinion about breastfeeding in the United States. *J Hum Lact*. 2005;21(3):284–8. 10.1177/0890334405278490 16113017

[38] Williams DR, Mohammed SA, Leavell J, Collins C. Race, socioeconomic status, and health: complexities, ongoing challenges, and research opportunities. *Ann N Y Acad Sci*. 2010;1186:69–101. 10.1111/j.1749-6632.2009.05339.x 20201869 PMC3442603

[39] Yudell M, Roberts D, DeSalle R, Tishkoff S. Taking race out of human genetics. *Science*. 2016;351(6273):564–5. 10.1126/science.aac4951 26912690

[40] Gee GC, Ford CL. Structural racism and health inequities: old issues, new directions. *Du Bois Rev*. 2011;8(1):115–32. 10.1017/S1742058X11000130 25632292 PMC4306458

[41] Hardeman RR, Medina EM, Kozhimannil KB. Structural racism and supporting black lives—the role of health professionals. *N Engl J Med*. 2016;375(22):2113–5. 10.1056/NEJMp1609535 27732126 PMC5588700

[42] Li R, Rock VJ, Grummer-Strawn L. Changes in public attitudes toward breastfeeding in the United States, 1999–2003. *J Am Diet Assoc*. 2007;107(1):122–7. 10.1016/j.jada.2006.10.002 17197280

[43] Nommsen-Rivers LA, Chantry CJ, Cohen RJ, Dewey KG. Comfort with the idea of formula feeding helps explain ethnic disparity in breastfeeding intentions among expectant first-time mothers. *Breastfeed Med*. 2010;5(1):25–33. 10.1089/bfm.2009.0052 20043707

[44] West E, Knight RJ. Mothers’ milk: slavery, wet-nursing, and black and white women in the antebellum South. *J South Hist*. 2017;83(1):37–68.

[45] Wong S-lC. Ch. 4: Divided mothering: representations of caregivers of color in the age of "multiculturalism". In: Glenn EN, Chang G, Forcey LR, editors. *Mothering*: *Ideology*, *Experience*, *and Agency*. New York, London: Routledge; 1994. p. 67–91.

[46] Pager D, Shepherd H. The sociology of discrimination: Racial discrimination in employment, housing, credit, and consumer markets. *Annu Rev Sociol*. 2008;34:181–209. 10.1146/annurev.soc.33.040406.131740 20689680 PMC2915460

[47] Tomaskovic-Devey D, Thomas M, Johnson K. Race and the accumulation of human capital across the career: A theoretical model and fixed-effects application. *Am J Sociol*. 2005;111(1):58–89.

[48] Wrigley-Field E, Seltzer N. Unequally insecure: rising black/white disparities in job displacement, 1981–2017. *Washington Center for Equitable Growth Working Paper Series*. Washington, DC. 2020.

[49] United States Bureau of Labor Statistics, Current Population Survey. *Labor force characteristics by race and ethnicity, 2018*. 2019.

[50] Golden L. Limited access: Disparities in flexible work schedules and work-at-home. *J Fam Econ Issues*. 2008;29(1):86–109.

[51] Hawkins D. Disparities in the usage of maternity leave according to occupation, race/ethnicity, and education. *Am J Ind Med*. 2020;63:1134‐44. 10.1002/ajim.23188 33020984

[52] Griswold MK, Crawford SL, Perry DJ, Person SD, Rosenberg L, Cozier YC, et al. Experiences of racism and breastfeeding initiation and duration among first-time mothers of the Black Women’s Health Study. *J Racial Ethn Health Disparities*. 2018;5(6):1180–91. 10.1007/s40615-018-0465-2 29435898 PMC6681652

[53] Ortiz SY, Roscigno VJ. Discrimination, women, and work: processes and variations by race and class. *Sociol Q*. 2009;50(2):336–59.

[54] Fekedulegn D, Alterman T, Charles LE, et al. Prevalence of workplace discrimination and mistreatment in a national sample of older U.S. workers: The REGARDS cohort study. *SSM Popul Health*. 2019;8:100444. 10.1016/j.ssmph.2019.100444 31321281 PMC6612926

[55] Dalianis EA. *Multi-level Analysis of the Role of the Workplace and Employment in Racial Disparities in Breastfeeding Practices*. PhD Dissertation, Drexel University. 2016. Available from: https://idea.library.drexel.edu/islandora/object/idea%3A6944

[56] Ahonen EQ, Fujishiro K, Cunningham T, Flynn M. Work as an inclusive part of population health inequities research and prevention. *Am J Public Health*. 2018;108(3):306–11. 10.2105/AJPH.2017.304214 29345994 PMC5803801

[57] Greenhaus JH, Beutell NJ. Sources of conflict between work and family roles. *Acad Manage Rev*. 1985;10(1):76–88.

[58] Hays S. *The Cultural Contradictions of Motherhood*. New Haven and London: Yale University Press; 1996.

[59] Afflerback S, Carter SK, Anthony AK, Grauerholz L. Infant-feeding consumerism in the age of intensive mothering and risk society. *Journal of Consumer Culture*. 2013;13(3):387–405.

[60] Dow DM. *Mothering While Black*: *Boundaries and Burdens of Middle-Class Parenthood*. Oakland, CA: University of California Press; 2019.

[61] Dow DM. Integrated motherhood: beyond hegemonic ideologies of motherhood. *Journal of Marriage and Family*. 2016;78(1):180–96.

[62] Collins C. *Making Motherhood Work*: *How Women Manage Careers and Caregiving*. Princeton, New Jersey: Princeton University Press; 2019.

[63] Dow DM. Negotiating “The Welfare Queen” and “The Strong Black Woman”: African American middle-class mothers’ work and family perspectives. *Sociol Perspect*. 2015;58(1):36–55.

[64] Herrick KA, Rossen LM, Kit BK, Wang C-Y, Ogden CL. Trends in breastfeeding initiation and duration by birth weight among US children, 1999–2012. *JAMA Pediatrics*. 2016;170(8):805–7. 10.1001/jamapediatrics.2016.0820 27367613 PMC6400210

[65] Office of the Surgeon General, Centers for Disease Control Prevention, Office on Women’s Health. *The Surgeon General’s Call to Action to Support Breastfeeding*. Rockville (MD); 2011.

[66] University of Michigan, Institute for Social Research. PSID Main Interview User Manual: Release 2019. February 2019.

[67] University of Michigan, Institute for Social Research, Survey Research Center. Panel Study of Income Dynamics. 2020. [Accessed 15 June 2020]. Available from: https://psidonline.isr.umich.edu/.

[68] MacDonald T, Noel-Weiss J, West D, Walks M, Biener M, Kibbe A, et al. Transmasculine individuals’ experiences with lactation, chestfeeding, and gender identity: a qualitative study. *BMC Pregnancy Childbirth*. 2016;16(1):106. 27183978 10.1186/s12884-016-0907-y PMC4867534

[69] United States Census Bureau. Industry and Occupation Code Lists & Crosswalks. 2020. [Accessed 21 October 2020] Available from: https://www.census.gov/topics/employment/industry-occupation/guidance/code-lists.html.

[70] Sullivan R. The age pattern of first-birth rates among US women: the bimodal 1990s. *Demography*. 2005;42(2):259–73. 10.1353/dem.2005.0018 15986986

[71] Xiang AH, Chow T, Mora-Marquez J, Martinez MP, Wang X, Yu W, et al. Breastfeeding persistence at 6 months: trends and disparities from 2008 to 2015. *J Pediatrics*. 2019;208:169–75.e2. 10.1016/j.jpeds.2018.12.055 30876751

[72] Yang Y, Morgan SP. How big are educational and racial fertility differentials in the US? *Soc Biol*. 2003;50(3–4):167–87. 10.1080/19485565.2003.9989070 16382810 PMC2849154

[73] Musick K, England P, Edgington S, Kangas N. Education differences in intended and unintended fertility. *Soc Forces*. 2009;88(2):543–72. 10.1353/sof.0.0278 23436948 PMC3578704

[74] Li R, Darling N, Maurice E, Barker L, Grummer-Strawn LM. Breastfeeding rates in the United States by characteristics of the child, mother, or family: the 2002 National Immunization Survey. *Pediatrics*. 2005;115(1):e31–e7. 10.1542/peds.2004-0481 15579667

[75] Fomby P, Cherlin AJ. Family instability and child well-being. *Am Sociol Review*. 2007;72(2):181–204. 10.1177/000312240707200203 21918579 PMC3171291

[76] Flacking R, Nyqvist KH, Ewald U, Wallin L. Long-term duration of breastfeeding in Swedish low birth weight infants. *J Hum Lact*. 2003;19(2):157–65. 10.1177/0890334403252563 12744532

[77] Acevedo-Garcia D, Soobader M-J, Berkman LF. The differential effect of foreign-born status on low birth weight by race/ethnicity and education. *Pediatrics*. 2005;115(1):e20–e30. 10.1542/peds.2004-1306 15629963

[78] Smith-Gagen J, Hollen R, Walker M, Cook DM, Yang W. Breastfeeding laws and breastfeeding practices by race and ethnicity. *Womens Health Issues*. 2014;24(1):e11–e9. 10.1016/j.whi.2013.11.001 24439936

[79] Settles IH, Pratt-Hyatt JS, Buchanan NT. Through the lens of race: Black and White women’s perceptions of womanhood. *Psychol Women Q*. 2008;32(4):454–68. 10.1111/j.1471-6402.2008.00458.x 29129954 PMC5679014

[80] Holmes AV, Auinger P, Howard CR. Combination feeding of breast milk and formula: evidence for shorter breast-feeding duration from the National Health and Nutrition Examination Survey. *J Pediatrics*. 2011;159(2):186–91. 10.1016/j.jpeds.2011.02.006 21429512

[81] McCluney CL, Rabelo VC. Conditions of visibility: An intersectional examination of Black women’s belongingness and distinctiveness at work. *J Vocat Behav*. 2019;113:143–52.

[82] Burgard SA, Lin KY. Bad jobs, bad health? How work and working conditions contribute to health disparities. *Am Behav Sci*. 2013;57(8):1105–27. 10.1177/0002764213487347 24187340 PMC3813007

[83] Braveman PA, Cubbin C, Egerter S, Williams DR, Pamuk E. Socioeconomic disparities in health in the United States: what the patterns tell us. *Am J Public Health*. 2010;100(S1):S186–S96. 10.2105/AJPH.2009.166082 20147693 PMC2837459

[84] Geyer S, Hemström Ö, Peter R, Vågerö D. Education, income, and occupational class cannot be used interchangeably in social epidemiology. Empirical evidence against a common practice. *J Epidemiol Community Health*. 2006;60(9):804–10. 10.1136/jech.2005.041319 16905727 PMC2566032

[85] Warren JR, Hoonakker P, Carayon P, Brand J. Job characteristics as mediators in SES–health relationships. *Soc Sci Med*. 2004;59(7):1367–78. 10.1016/j.socscimed.2004.01.035 15246167

[86] Hauser RM, Warren JR. Socioeconomic indexes for occupations: a review, update, and critique. *Sociol Methodol*. 1997;27(1):177–298.

[87] Davis R, Williams J, Chetwynd E. Increasing diversity in the field of lactation: an interview with the directors of Pathway 2 IBCLC programs at Historically Black Colleges and Universities. *J Hum Lact*. 2021;0(0):1–6. 10.1177/0890334421995108 33631997

[88] Walker-Tibbs N, Boyd L, Camara T, Brooks-Floyd F. The Black Course: Birth, Lactation, Accomodation, Culture, Kinship. 2020. [Accessed 6 Nov 2020]. Available from: https://theblackcourse.com/.

[89] Shipp G. Understanding Breastfeeding Self-Efficacy & Social Support in a Breastfeeding Duration Intervention Focused on African American Women. PhD Dissertation. Michigan State University 2020. Available from: https://search.proquest.com/docview/2449974935?pq-origsite=gscholar&fromopenview=true

[90] Dinour LM, Szaro JM. Employer-based programs to support breastfeeding among working mothers: a systematic review. *Breastfeed Med*. 2017;12(3):131–41. 10.1089/bfm.2016.0182 28394659

[91] Hilliard ED. A review of worksite lactation accommodations: Occupational health professionals can assure success. *Workplace Health Saf*. 2017;65(1):33–44. 10.1177/2165079916666547 27856993

[92] Spatz DL, Kim GS, Froh EB. Outcomes of a hospital-based employee lactation program. *Breastfeed Med*. 2014;9(10):510–4. 10.1089/bfm.2014.0058 25380384

[93] Moen P, Kelly EL, Tranby E, Huang Q. Changing work, changing health: can real work-time flexibility promote health behaviors and well-being? *J Health Soc Behav*. 2011;52(4):404–29. 10.1177/0022146511418979 22144731 PMC3267478

[94] Karasek RA Jr. Job demands, job decision latitude, and mental strain: Implications for job redesign. *Admin Sci Q*. 1979:285–308.

[95] Hagedorn J, Paras CA, Greenwich H, Hagopian A. The role of labor unions in creating working conditions that promote public health. *Am J Public Health*. 2016;106(6):989–95. 10.2105/AJPH.2016.303138 27077343 PMC4880255

[96] Finnigan R, Hale JM. Working 9 to 5? Union membership and work hours and schedules. *Social Forces*. 2018;96(4):1541–68.

[97] Kozhimannil KB, Attanasio LB, McGovern PM, Gjerdingen DK, Johnson PJ. Reevaluating the relationship between prenatal employment and birth outcomes: a policy-relevant application of propensity score matching. *Womens Health Issues*. 2013;23(2):e77–e85. 10.1016/j.whi.2012.11.004 23266134 PMC3596463

[98] Fabiyi C, Peacock N, Hebert-Beirne J, Handler A. A Qualitative study to understand nativity differences in breastfeeding behaviors among middle-class African American and African-born women. *Matern Child Health J*. 2016;20(10):2100–11. 10.1007/s10995-016-2029-6 27334637

[99] Goldberg AE, Smith JZ. Work conditions and mental health in lesbian and gay dual-earner parents. *Family Relat*. 2013;62(5):727–40.

