# Supplementary material for: Work, race and breastfeeding outcomes for mothers in the United States
Source: PLoS One. 2021 May 5;16(5):e0251125. doi: 10.1371/journal.pone.0251125 (PMC8099119; doi:10.1371/journal.pone.0251125)
Supplement: S1 Table — (DOCX) [file pone.0251125.s001.docx]

**S1 Table. Full set of multivariate models predicting breastfeeding initiation and duration by mother’s race and employment status/occupation type**

|  | **Minimal model:**  **Unadjusted effects of work and race**  **on breastfeeding** | | **Model 1:**  **Adjusted model,**  **no interaction term** | | **Model 2:**  **Adjusted model,**  **with interaction term** | |
| --- | --- | --- | --- | --- | --- | --- |
| **Odds of initiation** | **Ratio** | **95% CI** | **Ratio** | **(95% CI)** | **Ratio** | **(95% CI)** |
| Occupation type (compared to not working) |  |  |  |  |  |  |
| Professional/managerial | 1.582 | (0.917, 2.732) | 1.146 | (0.642, 2.046) | 0.942 | (0.463, 1.920) |
| Service/labor | 0.737 | (0.435, 1.252) | 0.747 | (0.438, 1.274) | 0.735 | (0.379, 1.425) |
| Race (White = Ref) |  |  |  |  |  |  |
| Black race | 0.469* | (0.299, 0.735) | 0.620 | (0.374, 1.028) | 0.641 | (0.278, 1.476) |
| Other race | 1.121 | (0.666, 1.890) | 1.294 | (0.750, 2.232) | 0.926 | (0.325, 2.638) |
| Occupation type * Race, interaction (White and not working = ref) |  |  |  |  |  |  |
| Black race & professional/managerial |  |  |  |  | 1.114 | (0.260, 4.784) |
| Black race & service/labor |  |  |  |  | 0.868 | (0.320, 2.359) |
| Other race & professional/managerial |  |  |  |  | 5.860 | (0.429, 79.967) |
| Other race & service/labor |  |  |  |  | 1.170 | (0.192, 7.142) |
| Age in years (range 15.6 to 45.8 years) |  |  | 0.988 | (0.942, 1.037) | 0.988 | (0.942, 1.036) |
| Education (bachelor’s degree or higher) |  |  | 2.119* | (1.203, 3.733) | 2.091* | (1.182, 3.701) |
| Mother is not married |  |  | 1.311 | (0.641, 2.680) | 1.296 | (0.632, 2.658) |
| Low birth weight infant |  |  | 0.807 | (0.322, 2.023) | 0.787 | (0.313, 1.979) |
| **Breastfeeding duration** | **Duration Ratio** | **95% CI** | **Duration Ratio** | **(95% CI)** | **Duration Ratio** | **(95% CI)** |
| Occupation type (Not working = ref) |  |  |  |  |  |  |
| Professional/managerial | 1.006 | (0.868, 1.166) | 0.953 | (0.815, 1.115) | 0.904 | (0.762, 1.072) |
| Service/labor | 0.816* | (0.688, 0.967) | 0.843* | (0.716, 0.993) | 0.795* | (0.663, 0.954) |
| Race (White = ref) |  |  |  |  |  |  |
| Black race | 0.690* | (0.593, 0.804) | 0.783* | (0.658, 0.933) | 0.562* | (0.444, 0.712) |
| Other race | 0.870 | (0.751, 1.008) | 0.865 | (0.744, 1.004) | 0.812 | (0.590, 1.118) |
| Occupation type * Race (White and not working = ref) |  |  |  |  |  |  |
| Black race & professional/managerial |  |  |  |  | 1.720* | (1.101, 2.686) |
| Black race & service/labor |  |  |  |  | 1.546* | (1.020, 2.343) |
| Other race & professional/managerial |  |  |  |  | 1.083 | (0.672, 1.746) |
| Other race & service/labor |  |  |  |  | 1.122 | (0.670, 1.877) |
| Age in years (range 15.6 to 45.8 years) |  |  | 1.022* | (1.007, 1.038) | 1.022* | (1.007, 1.037) |
| Education (bachelor’s degree or higher) |  |  | 1.002 | (0.881, 1.139) | 1.002 | (0.881, 1.139) |
| Mother is not married |  |  | 0.755* | (0.596, 0.957) | 0.758* | (0.597, 0.962) |
| Low birth weight infant |  |  | 0.702 | (0.489, 1.006) | 0.712 | (0.499, 1.014) |

*P-value <0.05

CI = Confidence Interval.

Models 1 and 2 are the same models shown Table 2 of main manuscript, but here the covariates are also included. All models are zero-inflated negative binomial models (ZINB) that include n=970 respondents. Coefficients displayed here have been exponentiated. The odds ratios in the top half of table represent the odds of initiating breastfeeding; we obtained these by exponentiating and then inverting the original coefficients from the ZINB model. Models included survey weights.
