# Supplementary material for: Work, race and breastfeeding outcomes for mothers in the United States
Source: PLoS One. 2021 May 5;16(5):e0251125. doi: 10.1371/journal.pone.0251125 (PMC8099119; doi:10.1371/journal.pone.0251125)
Supplement: S2 Table — (DOCX) [file pone.0251125.s002.docx]

**S2 Table. Predicted breastfeeding duration based on mother’s employment status/occupation type and race**

|  | **Model 1: Adjusted model without interaction term** | | | **Model 2: Adjusted model with interaction between race and employment status/occupation type** | | |
| --- | --- | --- | --- | --- | --- | --- |
|  | **Not working**  **Duration (95% CI)** | **Managerial/professional occupation**  **Duration (95% CI)** | **Service/labor occupation**  **Duration (95% CI)** | **Not working**  **Duration (95% CI)** | **Managerial/professional occupation**  **Duration (95% CI)** | **Service/labor occupation**  **Duration (95% CI)** |
| White race | 5.64 (4.91, 6.38) | 5.55 (4.52, 6.59) | 4.41 (3.65, 5.18) | 5.94 (5.15, 6.73) | 5.29 (4.16, 6.43) | 4.37 (3.40, 5.34) |
| Black race | 3.87 (3.02, 4.72) | 3.85 (2.81, 4.89) | 2.94 (2.13, 3.75) | 2.97 (2.13, 3.80) | 4.68 (2.28, 7.08) | 3.11 (2.23, 3.99) |
| Other race | 5.16 (4.01, 6.32) | 5.05 (4.23, 5.86) | 4.08 (3.14, 5.03) | 4.74 (3.20, 6.27) | 5.79 (4.06, 7.53) | 4.07 (2.43, 5.70) |

CI = Confidence Interval

The predicted margins for breastfeeding duration are based on two zero-inflated negative binomial regression models with n=970 respondents, both adjusted for potential confounders (mother’s age, educational attainment, marital status, and whether or not the infant was born with low birth weight). Model 1 predicted breastfeeding duration based on mother’s employment status/occupation type and race with no interaction term, while Model 2 included an interaction between race and employment status/occupation type.
