# Supplementary material for: Work, race and breastfeeding outcomes for mothers in the United States
Source: PLoS One. 2021 May 5;16(5):e0251125. doi: 10.1371/journal.pone.0251125 (PMC8099119; doi:10.1371/journal.pone.0251125)
Supplement: S3 Table — (DOCX) [file pone.0251125.s003.docx]

**S3 Table. Sensitivity analyses to assess effects of clustering among siblings born to same mother**

|  | **1. Breastfeeding initiation:**  **Not accounting for sibling clusters** | | **2. Breastfeeding initiation: Accounting for sibling clusters** | | **3. Breastfeeding duration:**  **Not accounting for sibling clusters** | | **4. Breastfeeding duration: Accounting for sibling clusters** | |
| --- | --- | --- | --- | --- | --- | --- | --- | --- |
|  | **OR** | **95% CI** | **OR** | **(95% CI)** | **b** | **(95% CI)** | **b** | **(95% CI)** |
| Occupation type (Not working = ref) |  |  |  |  |  |  |  |  |
| Professional/managerial | 1.059 | (0.616, 1.821) | 1.114 | (0.280, 4.436) | -0.981* | (-1.919, -0.044) | -0.993* | (-1.952, -0.034) |
| Service/labor | 0.702 | (0.437, 1.126) | 0.564 | (0.171, 1.856) | -1.770* | -(2.730, -0.811) | -1.461* | (-2.411, -0.512) |
| Race (White = ref) |  |  |  |  |  |  |  |  |
| Black race | 0.421* | (0.251, 0.706) | 0.123* | (0.031, 0.488) | -1.792* | (-3.005, -0.579) | -1.700* | (-2.910, -0.491) |
| Other race | 1.068 | (0.459, 2.486) | 1.171 | (0.146, 9.372) | -0.776 | (-2.421, 0.868) | -0.426 | (-2.064, 1.212) |
| Occupation type * Race (White and not working = ref) |  |  |  |  |  |  |  |  |
| Black race & professional/managerial | 1.456 | (0.622, 3.405) | 1.816 | (0.214, 15.441) | 1.963* | (0.187, 3.738) | 1.833 | (-0.005, 3.670) |
| Black race & service/Labor | 1.175 | (0.604, 2.285) | 0.781 | (0.150, 4.063) | 1.594 | (-0.006, 3.194) | 1.580* | (0.050, 3.111) |
| Other race & professional/managerial | 3.304 | (0.596, 18.324) | 11.326 | (0.299, 428.695) | -0.499 | (-2.752, 1.755) | -0.793 | (-3.067, 1.481) |
| Other race & service/labor | 0.888 | (0.297, 2.659) | 0.660 | (0.044, 9.938) | 0.871 | (-1.388, 3.129) | 0.289 | (-1.911, 2.489) |

OR = Odds ratio. b= Regression coefficient.

*P-value <0.05

The models for breastfeeding initiation (models 1 and 2) were based on logistic regression with n= 969 respondents. The models for breastfeeding duration (3 and 4) were based on multivariate linear regression models with n=627 respondents. The two models that control for clustering among siblings (2 and 4) were mixed models with random effects the mother. Models 1 and 3 are similar to the initiation and duration components of the interaction model shown in Table 2 of the main manuscript, but they are not identical because the models in Table 2 were estimated using a different modeling approach.
